# Conformational Control of Chemical Reactivity for Surface‐Confined Ru‐Porphyrins

**DOI:** 10.1002/anie.202104075

**Published:** 2021-06-22

**Authors:** Peter Knecht, Joachim Reichert, Peter S. Deimel, Peter Feulner, Felix Haag, Francesco Allegretti, Manuela Garnica, Martin Schwarz, Willi Auwärter, Paul T. P. Ryan, Tien‐Lin Lee, David A. Duncan, Ari Paavo Seitsonen, Johannes V. Barth, Anthoula C. Papageorgiou

**Affiliations:** ^1^ Physics Department E20 Technical University of Munich James Franck Straße 1 85748 Garching Germany; ^2^ Current address: Instituto Madrileño de Estudios Avanzados en Nanociencia (IMDEA-Nanociencia), Cantoblanco 28049 Madrid Spain; ^3^ Diamond Light Source Didcot OX11 0DE UK; ^4^ Department of Materials Imperial College London Exhibition Road SW7 2AZ London UK; ^5^ Current address: Institute of Applied Physics Technische Universität Wien Wiedner Hauptstraße 8-10/134 1040 Vienna Austria; ^6^ Département de Chimie Ecole Normale Supérieure 24 rue Lhomond 75005 Paris France; ^7^ Université de recherche Paris-Sciences-et-Lettres Sorbonne Université Centre National de la Recherche Scientifique 75005 Paris France

**Keywords:** ab-initio calculations, CO ligands, metalloporphyrins, scanning probe microscopy, X-ray spectroscopy

## Abstract

We assess the crucial role of tetrapyrrole flexibility in the CO ligation to distinct Ru‐porphyrins supported on an atomistically well‐defined Ag(111) substrate. Our systematic real‐space visualisation and manipulation experiments with scanning tunnelling microscopy directly probe the ligation, while bond‐resolving atomic force microscopy and X‐ray standing‐wave measurements characterise the geometry, X‐ray and ultraviolet photoelectron spectroscopy the electronic structure, and temperature‐programmed desorption the binding strength. Density‐functional‐theory calculations provide additional insight into the functional interface. We unambiguously demonstrate that the substituents regulate the interfacial conformational adaptability, either promoting or obstructing the uptake of axial CO adducts.

## Graphical abstract

The CO ligation to two different ruthenium porphyrins on the Ag(111) surface under vacuum was investigated experimentally and theoretically. The electronic and geometric changes upon ligation as well as the energetics of the process were elucidated. The surface adaptability of the porphyrin macrocycle conformation proves crucial for CO binding.

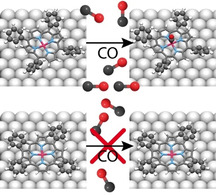

## Introduction

For the creation of novel materials and devices, inspiration is frequently sought in nature. Porphyrins and other natural tetrapyrrole compounds can incorporate a large fraction of the chemical elements in the periodic table. Their functionality is tuned by choice of the complexed species, possible axial ligands and substituents in the macrocycle periphery. For example, in biology, the binding of small molecules to metal centres determines many vital functions. Over the past decades we have witnessed an intense interest in utilizing porphyrins on surfaces as functional building blocks with a myriad of possible applications: ranging from atomic switches to single‐molecule magnets and catalysts.^[1]^ The surface chemistry of cyclic tetrapyrrole compounds is therefore a topic of extended research^[2]^ and includes the on‐surface metallation^[3]^ as well as s‐block^[4]^ and p‐block^[5]^ element functionalisation. In this context, the effect of the macrocycle substituents has been studied systematically.^[6]^ Moreover, the reactivity of individual metal atoms on surfaces is a topical issue in single‐atom catalysis,^[7]^ whereby arrays of metalloporphyrin layers under vacuum conditions present a versatile playground due to the coordinatively unsaturated metal centres provided by the generally favoured adsorption geometries with the macrocycle residing parallel to the substrate lattice.

**Figure 1:**
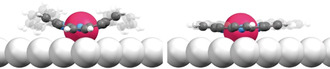
Models of a Ru tetraphenyl porphyrin (Ru‐TPP, left) and a planarized Ru‐TPP derivative (right) on Ag(111). The substituents are faded to highlight the difference in the conformation of the porphyrin macrocycles.^[20]^ Ru, C, N, H, and Ag are shown in raspberry, grey, blue, white, and silver, respectively.

Complexes of inorganic gaseous molecules with metalloporphyrins are important intermediate species in catalysis. CO,^[8]^ NO,[^8a^, ^9^] NH_3_,^[10]^ and H_2_O^[10]^ have been shown to bind on metal supported metalloporphyrins and phthalocyanines axially to the metal centre. In particular, CO also exhibited an unusual *cis*‐μ‐dicarbonyl ligation on top of Fe (and Co) tetraphenyl porphyrins on Ag(111) (and Cu(111)).^[11]^ Ligation to the metal centre gives rise to the so‐called structural *trans*‐effect, whereupon the metal atom is electronically and physically decoupled from the substrate.[^9^, ^10^, ^12^] Generally, a significant alteration of the porphyrin's reactivity and electronic structure occurs due to the interaction with the metal surface.^[13]^ Turning our attention on the topic of “*switch on*” functionalities of organic layers on metal surfaces, we can find a common approach of “decoupling” the molecule from the surface by for example, a rigid tethering,^[14]^ bulky substituents,^[15]^ or a platform^[16]^ which enables a “lift‐off” of the functional moiety. In a biological environment, the macrocycle conformation can influence its functionality.^[17]^ Here, we will examine this aspect: can we influence the function present in the free molecule (here CO binding) by the conformation of the porphyrin macrocycle (Figure 1), hosting the metal centre, on the surface?

For Ru tetraphenyl porphyrins (Ru‐TPPs), CO is determined to have an unusually high ligation energy (1.9 eV),^[18]^ hence can be considered as a prototypical out‐of‐plane ligand with the stability of a covalent attachment. Here, we study the effect of the porphyrin surface environment on this ligation for Ru‐TPP and its planarized derivatives (Ru‐TPP_pl_) on Ag(111). We use scanning tunnelling microscopy (STM) to find a *cis*‐μ‐dicarbonyl ligation^[11]^ stable at low temperatures (5 K) and an axial ligation at higher temperatures (200 K), which is also examined with temperature programmed desorption (TPD). In stark contrast, there is no evidence of CO binding to the planarized Ru‐TPP derivatives on Ag(111) under either conditions. We correlate the axial binding to conformational and electronic changes, rationalised by density functional theory (DFT), X‐ray and ultraviolet photoelectron spectroscopy (XPS and UPS) and normal incidence X‐ray standing waves (NIXSW).

## Results and Discussion

### Imaging in Real Space

**Figure 2:**
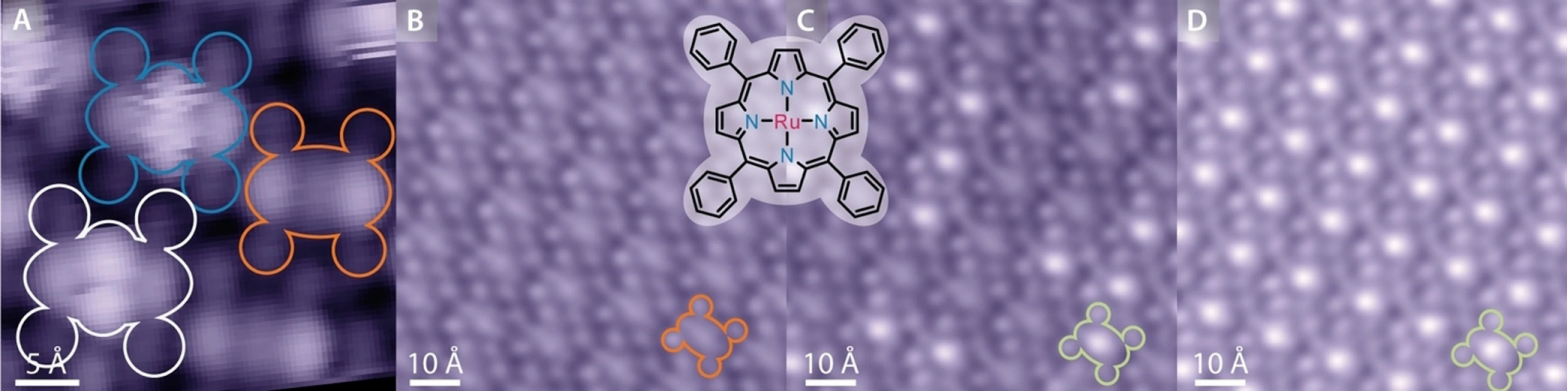
STM images of CO‐ligation on Ru‐TPP on Ag(111). A) Ligation modes at 5 K: μ‐geometry (white & blue) and uncapped (orange). (−1.0 V, 50 pA) B–D) Evolution of Ru(CO)‐TPP formation (capped molecules marked in green) in situ at 150 K under CO exposure (1.25 V, 80 pA).

When deposited on Ag(111) at room temperature in submonolayer coverages, Ru‐TPP (Figure 2 inset) molecules self‐assemble in a square phase^[19]^ described by the epitaxial matrix $\left(\begin{array}{cc} 7 & 0\\ 4 & 8 \end{array}\right)$ .^[20]^ Figure 2 A shows the assembly on such a surface cooled down to 5 K (overview image in Figure S1). For negative bias voltages (≈−1 V), the single molecule appearance of the pristine Ru‐TPP (outlined in orange) is characterised by three bright protrusions along the macrocycle and four less bright in the periphery marking the phenyl substituents. The central bright protrusion corresponds to a filled electronic state of the Ru centre (cf. UPS below),^[21]^ whereas the outer ones can be assigned to the protruding άνω‐pyrroles (α‐pyr) of the macrocycle.^[11]^ The downward bending κάτω‐pyrroles (κ‐pyr) are not discernible. In the STM images of the layer we can also identify molecules with additional protrusions, located on the sides of the Ru centre and perpendicular to the axis of the α‐pyr (examples outlined in white and blue). Their STM appearance is virtually identical to the μ‐carbonyl rider ligation on Co‐TPP and Fe‐TPP,^[11]^ and given a small residual pressure of CO (cf. experimental section), we can confidently assign those to the analogous Ru‐TPP ligation. The molecule outlined in white can be identified as featuring a *cis*‐μ‐dicarbonyl binding geometry (Figure S2) and the example outlined in blue is characteristic of a single CO adsorbed in the rider mode and switching between the two adsorption sides during the STM imaging.

Performing STM investigations at higher temperatures (150 K), we found solely a single mode of CO ligation, recognisable by uniform protrusions directly on top of the Ru centres (example outlined in green in Figure 2 C,D). We attribute these to axial carbonyls.^[8c]^ Monitoring the same area of a Ru‐TPP layer by STM (Figure 2 B–D), while dosing CO in situ, we note that increasing the CO exposure led to an increase in the number of protrusions until all Ru‐TPP molecules became brighter, which was achieved after a nominal exposure to ≈2 Langmuir (L) of CO. It should be noted that an estimate of the sticking coefficient cannot be extrapolated from the nominal value, as the real exposure will differ due to effects such as tip shadowing.

**Figure 3:**
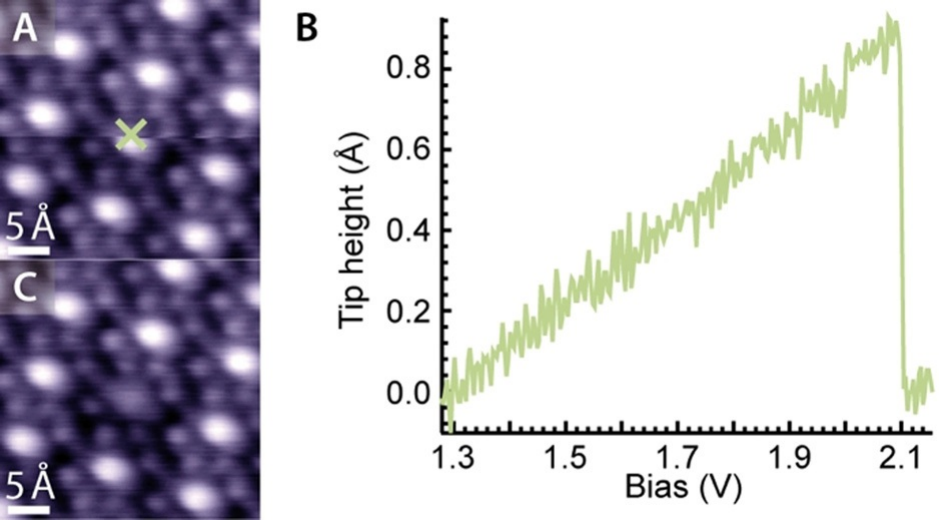
Removal of a single CO ligand by a voltage pulse with the STM tip. A) While recording the STM image (1.25 V, 50 pA, scanning from bottom to top), a voltage pulse was applied at the position marked by the green cross. B) Tip height profile of the voltage pulse from 1.28 V to 2.15 V, with a current of 50 pA. At ≈2.1 V the tip approaches the surface abruptly, indicating desorption of CO. C) Follow‐up STM image (1.25 V, 50 pA) confirming the removal of the ligand.

The CO ligands can be selectively removed by STM tip manipulations at 150 K as illustrated in the sequence of STM images in Figure 3: At the position marked by the green cross (Figure 3 A), the voltage was ramped from 1.28 V to 2.15 V in a constant current mode (50 pA) while monitoring the tip height (Figure 3 B). A sudden change in the vertical tip position at ≈2.1 V indicates desorption of the CO ligand, which is confirmed by a follow‐up image revealing a pristine Ru‐TPP at the location of the voltage pulse (Figure 3 C). This procedure allows reliable removal of single CO ligands. On similar systems both tunnelling current induced desorption[^8c^, ^22^] and electric field induced desorption^[23]^ have been observed, though smaller bias voltages (<1 V) were required. For voltages >1.5 V, a non‐local desorption is often reported.[^8b^, ^22^, ^24^] Such a desorption behaviour is also observed in this system with a bias voltage of 2.0 V, when higher tunnelling currents are applied (Figure S3).

**Figure 4:**
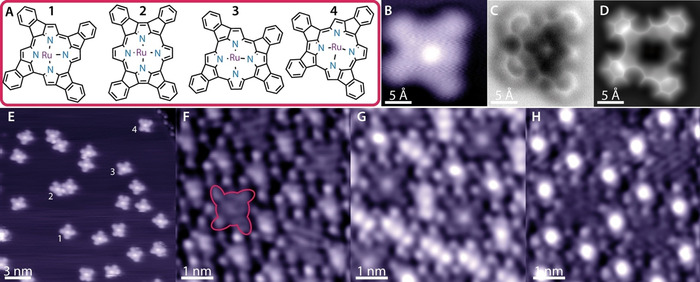
A) Structural formulas of the planarized Ru‐TPP derivatives (Ru‐TPP_pl_). B–D) Images of the Ru‐TPP_pl_ **4** on Ag(111). B) STM topography (−50 mV, 50 pA, 5 K). C) nc‐AFM frequency shift image (tip height Z=−20 pm with respect to the STM set point above Ag(111), oscillation amplitude 80 pm, 5 K). D) nc‐AFM simulation. E) STM overview image (−0.5 V, 50 pA) of the mixture of Ru‐TPP_pl_ after CO exposure at 5 K. The bright central protrusion arises from the Ru centre. The numbers (1–4) indicate examples of the four different derivatives (**1**–**4**), respectively. F–H)  Mixed layer of Ru‐TPP_pl_ product **1** and Ru‐TPP before (F), during (G) and after (H) CO exposure at 150 K (F,H: 1.25 V, 50 pA, G: −1.25 V, 50 pA, see Figure S5 for an identification of the different species).

To investigate the effect of the macrocyclic conformation on the CO ligation to the Ru centre, we have investigated the adsorption behaviour of Ru‐TPP on Ag(111) after annealing to 620 K. This process causes cyclodehydrogenation reactions between the macrocycle periphery and the phenyl substituents, leading to a family of four planarized Ru‐TPP derivatives, Ru‐TPP_pl_.[^19^, ^20^] Based on DFT calculations, the binding energy of the most commonly occurring Ru‐TPP_pl_ **3** ^[19]^ to Ag(111) is 5.66 eV, 1.26 eV higher than that of pristine Ru‐TPP. The different derivatives can be identified by matching the characteristic outline of the structural formula (Figure 4 A) to the STM image (Figure 4 B,E), whereas nc‐AFM imaging can visualise more directly the chemical identity, as illustrated for one of the more frequently occurring species in Figure 4 C. The resulting porphyrin macrocycle appears to exhibit a subtle bowl shape with pyrrole tilt angles of 6° and 8° (see nc‐AFM and respective simulation in Figure 4 C,D and Table 2) and also offers a coordinatively unsaturated metal centre. We note that the surface depicted in Figure 4 E has been exposed to the small amounts of CO at 5 K needed for the tip functionalisation, however no evidence of a lateral adsorbate stabilisation was found on the Ru‐TPP_pl_ molecules by STM/nc‐AFM. As the rider ligation is associated with the saddle shape deformation,^[11]^ we would not expect this bowl configuration to permit such ligation. However, it is with some surprise that we do not observe an axial ligation at all. The protrusion in the centre of Ru‐TPP_pl_ observed in STM at negative bias (Figure 4 B,E,G) arises, similarly to Ru‐TPP, from the Ru centre^[21b]^ (cf. UPS below) and is not related to potential CO adsorption, as confirmed by the corresponding nc‐AFM image (Figure 4 C). At experiments of methodical exposure of Ru‐TPP_pl_/Ag(111) to CO at 150 K (Figure 4 F–H and Figure S4) no CO uptake by Ru‐TPP_pl_ was observed in STM data.

To corroborate the difference in adsorption behaviour clearly with the same STM tip conditions, we prepared a sample containing both Ru‐TPP and Ru‐TPP_pl_ molecules on Ag(111) (Figure 4 F). In this mixed lattice exclusively the planarized derivative **1** appears (Figure 4 A). This effect is associated with the different molecular shapes of other product species that do not fit into the expressed overlayer lattice (Figure S5).^[25]^ The (stepwise) exposure of this layer to doses of CO at 150 K resulted in saturating exclusively all Ru‐TPP centres with CO (Figure 4 H), whereas no changes were encountered for the Ru‐TPP_pl_ species. An intermediate CO coverage acquired at negative bias (Figure 4 G) highlights the difference in STM appearance between Ru‐TPP, Ru(CO)‐TPP and Ru‐TPP_pl_ (see also Figure S5).

### Binding Energy and Desorption Kinetics

**Figure 5:**
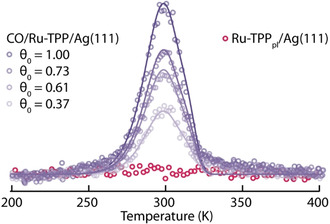
Coverage dependant TPD spectra and fitting of CO desorption for *m*/*z*=28. Different shades of purple indicate different initial CO coverages *θ*_0_, dosed at 200 K, on the same Ru‐TPP layer. A heating rate of 2 K s^−1^ was used. The red spectrum shows the same trace for Ru‐TPP_pl_ after CO exposure, confirming that CO is not ligating.

To deduce information about the bond strength of the axially ligated CO on the Ru‐TPP layer and to confirm that CO does not ligate to the Ru‐TPP_pl_ under the same conditions, we carried out systematic TPD measurements. After exposure of the square phase of Ru‐TPP on Ag(111) to CO, our results show exclusively CO desorption (Figure S6) in the temperature range of 200–550 K. Dosing different amounts of CO onto a layer of Ru‐TPP has no effect on the shape of the desorption curve, but only on the intensity (Figure 5, purple), indicative of first order desorption kinetics. The acquired spectra can be modelled by assuming a pre‐exponential factor of *ν*=10^13^ s^−1^ and including two first‐order desorption processes of equal intensities with energies of *E*_des,1_=0.80 eV and *E*_des,2_=0.84 eV (see details in Supporting Information and Figure S7). The difference in binding energy of 0.04 eV could be related to Ru(CO)‐TPP adsorption on both *fcc* and *hcp* hollow sites of the Ag(111) surface (see DFT model of optimised structure in Figure S8). We note that consistent with our experiments, in such a case we would not expect a preferential occupation for the lower binding adsorption site, as no exchange of CO between the molecules is possible at 200 K and the desorption temperature from the Ag(111) is much lower.^[26]^ However, we cannot exclude a more complex desorption behaviour as a cause for the spectra's signature. While the desorption energy is very comparable to values found for Ru(CO)‐TPP on the more reactive Cu(110) surface,^[8c]^ it is significantly smaller compared to gas‐phase molecules.^[18]^ After exposing a layer of Ru‐TPP_pl_ to CO, there is no desorption trace of CO detected (Figure 5, red), confirming the results from STM/AFM measurements that CO is not ligating to Ru‐TPP_pl_.

At this stage, the following two questions arise: (1) How is the Ru‐TPP affected by the CO ligation? (2) Why are these very similar porphyrins so different in their chemical reactivity? The following analysis will discuss the impact of the CO ligation on electronic and geometric properties of the Ru‐TPP.

### Electronic Structure

**Figure 6:**
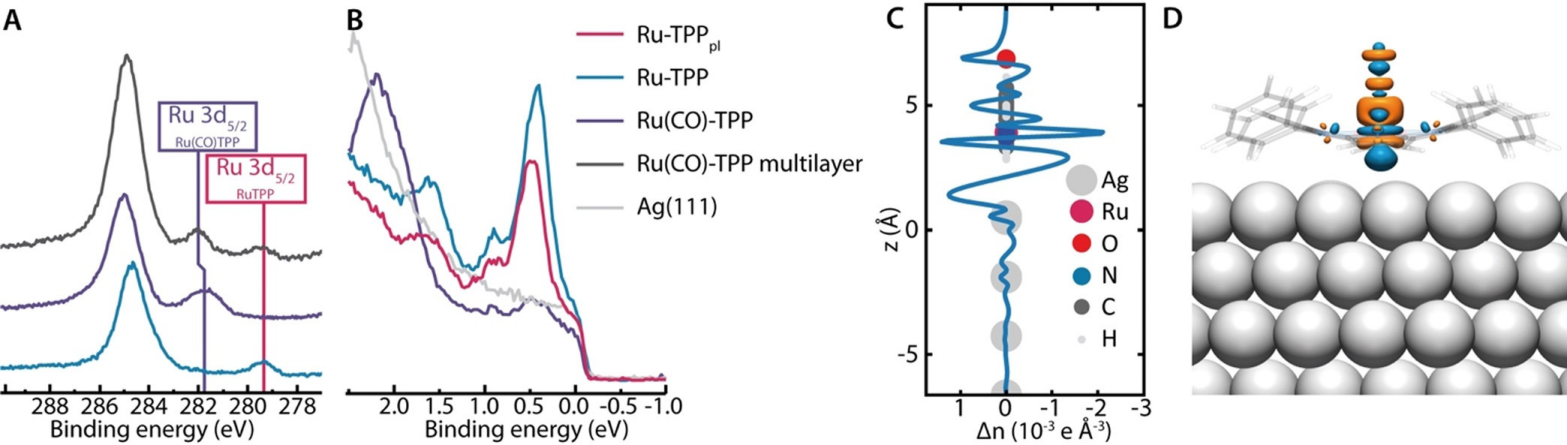
A) XP spectra of the C 1s & Ru 3d region corresponding to (from bottom to top) submonolayer coverages of pristine Ru‐TPP (300 K), Ru(CO)‐TPP (80 K) and a multilayer of Ru(CO)‐TPP (300 K) on Ag(111). B) UP spectra for clean Ag(111) (grey), pristine Ru‐TPP (blue), Ru(CO)‐TPP (purple) and Ru‐TPP_pl_ (red) on Ag(111). C,D) Charge density redistribution upon CO adsorption on Ru‐TPP on Ag(111) deduced from DFT. The one dimensional plot (C) shows differences in the electron density normal to the Ag(111) surface upon CO ligation, the three dimensional plot (D) shows isosurfaces (0.04 e Å^−3^) indicating gain of electron density (orange) and loss of electron density (blue).

We initially investigated the XPS signature of the Ru 3d_5/2_ core level as a measure of the electronic interaction with the metal substrate (Figure 6 A). Upon ligation of CO, the binding energy of the Ru 3d_5/2_ core level shifts by 2.4 eV towards higher binding energies, indicating a decoupling of the Ru centre from the Ag substrate. The shift towards higher binding energies is in good accord with the DFT prediction (+1.8 eV). Note that the Ru 3d_3/2_ component is coincident with the C 1s peak (≈285 eV), and can be observed as a small shoulder on the lower binding energy side for Ru‐TPP and at the higher binding energy side for Ru(CO)‐TPP layers.^[21a]^

From the XPS of a multilayer of Ru(CO)‐TPP on Ag(111) one can deduce that the CO ligand remains attached to the Ru‐TPP on the layers without direct contact to the Ag(111) substrate at 300 K.^[27]^ The binding energy of the Ru 3d_5/2_ core level of Ru(CO)‐TPP directly on Ag(111) is 0.2 eV lower than that observed in the multilayer films. Such a shift is consistent with the expected polarisation screening by the metal substrate.

UP spectra further show states for both Ru‐TPP (Figure 6 B, blue) and Ru‐TPP_pl_ (Figure 6 B, red) at binding energies of 0.4 eV and 0.9 eV, which can be correlated to the bright protrusion at negative bias voltages in the STM images (Figure 2 A,B,E,G), similar to Co‐TPP on Ag(111).^[28]^ These Ru states are extinguished for Ru(CO)‐TPP (Figure 6 B, purple), indicating that the interaction of Ru centres and the Ag substrate, responsible for these states, is no longer present upon ligation (Figure S9).^[9]^

Further insight into the electronic changes upon the adsorption of CO is gained from DFT. Figure 6 C shows that changes in the electron density upon CO adsorption are not simply restricted to the porphyrin, but also evident in the Ru‐TPP/Ag(111) interface. The charge at the interface per molecule is significantly reduced upon CO ligation (Δ*q*_Int_=−0.47 *e*, *q*_Int_=0.18 *e*), confirming the electronic decoupling of the Ru‐TPP molecules from the Ag(111) surface correlated to the CO ligation and the surface *trans*‐effect. While the CO is negatively charged (*q*_CO_=−0.18 *e*), the Ru centre gets more positively charged (Δ*q*_Ru_=0.27 *e*). A closer inspection of the orbital structure (Figure 6 D) reveals a decreased electron density in the 5σ orbitals of the CO and a commensurate increase in electron density in the 2π orbitals, in agreement with the Blyholder model for chemisorbed carbon monoxide.^[29]^ On the ruthenium centre, a decrease of electron density in the $d_{z^{2}}$ orbital, as well as an increase in the d_zx_ and d_yz_ orbitals is observed. It is notable that this change in the Ru 3d electron density is similar between, both, the Ru—CO, and Ru—Ag, whereas for Ru‐TPP_pl_ **3** the corresponding DFT calculations find the electron accumulation to be in $d_{z^{2}}$ , and depletion in the d_*zx*_, d_*yz*_ orbitals.^[20]^ The depletion and gain of electrons in orbitals of both Ru and CO show a back‐donation of electrons from the Ru centre to the CO ligand, which in addition to the decoupling can contribute to the increase in binding energy of the Ru 3d_5/2_ core level upon CO ligation (Figure 6 A).

### Structural Determination

**Figure 7:**
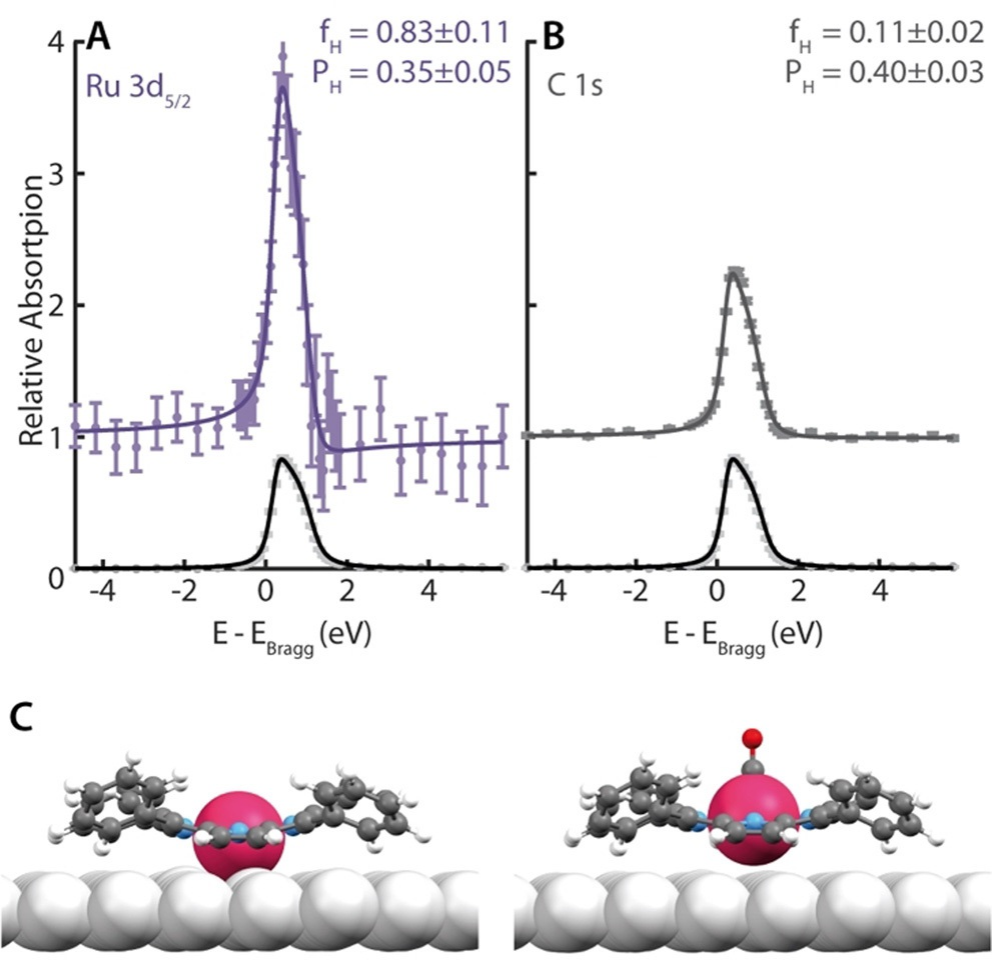
A,B) NIXSW photoelectron profiles and fits of the Ru 3d_5/2_ and C 1s regions in (111) reflection for Ru(CO)‐TPP. Purple (dark grey) dots indicate the Ru (C) data points, light grey dots the reflection of the Ag(111) substrate. C) DFT model of Ru‐TPP^[20]^ (left) and Ru(CO)‐TPP (right) on Ag(111). Ru, Ag, C, N, O and H atoms are depicted in raspberry, silver, grey, blue and white, respectively.

Our earlier structural investigation of Ru‐TPP and Ru‐TPP_pl_ has shown that the adsorption height of the Ru centre differs only by 0.14 Å.^[20]^ Nevertheless, the adsorption height of the Ru centre of Ru‐TPP increases upon CO ligation at 200 K by 0.59 Å from 2.59±0.05 Å^[20]^ to 3.18±0.12 Å, as shown by NIXSW data of the Ru(CO)‐TPP (Figure 7 A, Table 2).^[30]^ The high coherent fraction indicates a very well‐defined adsorption height for the molecules, confirming a rather uniform geometry.^[31]^ The C 1s NIXSW data (Figure 7 B, Table 2) show an increased average adsorption height also for the carbon atoms. Thus, we conclude that the non‐planarity of the Ru‐TPP facilitates a conformational change of the entire Ru‐TPP upon CO ligation and enables the decoupling of the Ru centre from the Ag surface. One should note that the NIXSW measurements could only be performed on mixed layers of Ru(CO)‐TPP and pristine Ru‐TPP. While the Ru 3d_5/2_ peaks of the two species can be clearly distinguished due to the large shift in binding energy (Figure 6 A, Table 1), allowing the adsorption height for each species to be analysed individually, this is not possible for the C 1s. The carbon spectra, as described above, have to be understood as an average over all carbon atoms from both species, which includes additionally a negligible contribution of the Ru 3d_3/2_ core level. Therefore, only a qualitative comparison is meaningful. With this in mind, the XSW results are in excellent agreement with complementary DFT calculations (Figure 7 C, Table 2), which predict an increase of the Ru adsorption height of 0.69 Å and an increase of the average C adsorption height of 0.11 Å. We propose that the porphyrin macrocycle is lifted, while the phenyl substituents remain in contact with the Ag(111) substrate. These conformational adaptations can be interpreted as a rather strong surface *trans*‐effect.[^10^, ^12a^, ^32^]

**Table 1:** XPS binding energies of Ru 3d, C 1s & N 1s core levels for a submonolayer coverage of Ru‐TPP on Ag(111), a submonolayer coverage of Ru(CO)‐TPP on Ag(111) and Ru(CO)‐TPP on Ru‐TPP monolayer on Ag(111).

		Ru‐TPP/Ag(111) [eV]	Ru(CO)‐TPP/Ag(111) [eV]	Ru(CO)‐TPP/Ru‐TPP/Ag(111) [eV]
Ru 3d_5/2_	Ru‐TPP	279.4±0.1		279.4±0.1
	Ru(CO)‐TPP		281.8±0.1	282.0±0.1
C 1s		284.7±0.1	285.0±0.1	284.9±0.1
N 1s		398.6±0.1	398.9±0.1	398.8±0.1

**Table 2:** Structural parameters by DFT and NIXSW results from Ru 3d_5/2_ and C 1s core levels for the different investigated systems. The adsorption heights are deduced from the coherent position, for the C atoms it is an average value. In parentheses, we report the DFT simulated adsorption height that would be the result of the respective NIXSW measurement for comparison.

DFT	Pyrrole (α‐pyr/κ‐pyr) tilt angle by DFT	Adsorption height [Å] by DFT		Adsorption height [Å] by NIXSW	
		Ru	C	Ru	C
Ru‐TPP^[20]^	28°/−9°	2.68	3.53 (3.20)	2.59±0.05	3.02±0.07
Ru(CO)‐TPP	23°/−14°	3.37	3.64 (3.44)	3.18±0.12	3.30±0.07
Ru‐TPP_pl_ ^[20]^	8°, 6°	2.48	3.01 (3.01)	2.45±0.02	2.99±0.05
Ru(CO)‐TPP_pl_	5°, 0°	2.97	2.99 (2.99)

To understand the anticipated structural *trans*‐effect for planarized Ru(CO)‐TPP derivatives, we investigated a DFT geometry optimisation (Figure S10). Here, the *trans*‐effect would increase the Ru adsorption height by 0.49 Å whereas it would leave the macrocycle mostly unaffected (Table 2). In comparison with the saddle‐shaped pristine TPP, these deformations are smaller and show less adaptation of the macrocycle with CO ligation, which is more restricted by its adsorption to the silver surface.

It is notable that the planarized Ru(CO)‐TPP derivative investigated is also a stable geometry in simulation with a binding energy of the CO predicted to be smaller by 0.5 eV with respect to the pristine Ru‐TPP. We can thus attribute the lack of experimental evidence of this species to either a higher activation barrier associated with the decoupling of the Ru from the silver surface or to CO sticking coefficient differences of more than an order of magnitude.

## Conclusion

We have studied the CO ligation on distinct Ru‐porphyrins on a silver surface by a combined theoretical and experimental analysis of the electronic and geometric effects of such a ligation.

Rider CO‐ligation at low temperatures (at 5 K) and axial CO‐ligation (up to ≈250 K), in agreement with the Blyholder model^[29]^ for chemisorption, were observed only for the pristine saddle‐shape Ru‐TPP. STM allowed tip induced desorption of single ligands without damaging the Ru‐TPP underneath, which can be used to create patterns on a nanometer scale. The large shift in binding energy of the Ru 3d_5/2_ core level upon axial ligation indicates an electronic decoupling of the Ru centre from the surface and both NIXSW and DFT have confirmed significant conformational changes. While the Ru centre is affected the most, increasing in adsorption height by ≈0.6 Å, an increase of the adsorption height is observed for the entire molecule (Figure 7 C). From TPD we determined the desorption energy of the axial CO ligand to be 0.8±0.1 eV, reduced by 1.1 eV in comparison to the CO binding strength to the free Ru‐TPP.

For the planarized Ru‐TPP derivatives, there was no sign of CO ligation in STM, AFM and TPD measurements. With the bonding of the Ru centre to the Ag surface being similar for both investigated porphyrins, our results emphasize the crucial role of the flexibility of the Ru‐TPP in the ligation process and the related ease of decoupling of the Ru centre from the Ag(111) surface.

Our findings with this model Ru‐porphyrin/Ag(111) system are expected to be relevant for the elucidation to processes related to gas sensing,^[33]^ and to supported single‐atom catalysts (e.g. Ru‐N4).

## Conflict of interest

The authors declare no conflict of interest.

## Supporting information

As a service to our authors and readers, this journal provides supporting information supplied by the authors. Such materials are peer reviewed and may be re‐organized for online delivery, but are not copy‐edited or typeset. Technical support issues arising from supporting information (other than missing files) should be addressed to the authors.

Supplementary
