# Supplementary material for: Conformational Control of Chemical Reactivity for Surface‐Confined Ru‐Porphyrins
Source: Angew Chem Int Ed Engl. 2021 Jun 22;60(30):16561–7. doi: 10.1002/anie.202104075 (PMC8362151; doi:10.1002/anie.202104075)
Supplement: Supplementary file 1 — Supplementary [file ANIE-60-16561-s001.pdf]

Angewandte Chemie
Eine Zeitschrift der Gesellschaft Deutscher Chemiker

Supporting Information

Conformational Control of Chemical Reactivity for Surface-Confined Ru-Porphyrins

Peter Knecht, Joachim Reichert, Peter S. Deimel, Peter Feulner, Felix Haag, Francesco Allegretti, Manuela Garnica, Martin Schwarz, Willi Auwärter, Paul T. P. Ryan, Tien-Lin Lee, David A. Duncan, Ari Paavo Seitsonen, Johannes V. Barth, and Anthoula C. Papageorgiou**

anie_202104075_sm_miscellaneous_information.pdf

Supporting Information

Table of Contents

Experimental Procedures **1**

Sample preparation STM/AFM/NIXSW 1

Sample preparation XPS/UPS/TPD measurements 2

DFT calculations 2

Results **4**

CO ligation on Ru-TPP at 5 K 4

Cis-µ-carbonyl bonding 4

STM tip manipulation for non-local CO desorption 4

Planarized Ru-TPP on Ag(111) 5

Mixed layer of Ru-TPP and Ru-TPP$_{pl}$ on Ag(111) 5

2D-TPD spectra of CO desorption from Ru-TPP/Ag(111) 5

TPD analysis of CO desorption from Ru-TPP 6

Fitting of TPD spectra for CO desorption from Ru-TPP 6

DFT model of Ru(CO)-TPP on Ag(111) 7

Density of states of the Ru centre for Ru-TPP, Ru-TPP$_{pl}$ and Ru(CO)-TPP 7

DFT model of Ru(CO)-TPP$_{pl}$ 8

References **8**

Experimental Procedures

Sample preparation STM/AFM/NIXSW

The samples were prepared under ultra-high vacuum (UHV) conditions. The atomically flat and clean Ag(111) single crystal surfaces were prepared by multiple cycles of Ar+-sputtering and annealing at 725 K.
Ru(CO)-TPP (Sigma Aldrich, 80% dye content) was used for dosing Ru-TPP by organic molecular beam epitaxy (OMBE, commercial source from DODECON nanotechnology GmbH).[1] The heating temperature for Ru(CO)TPP was 605-625 K, which resulted in Ru-TPP deposition rates of 0.3 - 0.6 molecules/(nm²·h) on the Ag(111) crystal held at room temperature.
Ru-TPP molecules were converted to their planarized derivatives by annealing at 620 K. Cyclodehydrogenation reactions between the macrocycle and the phenyl substituents result in a characteristic distribution of planarized derivatives.[2]
The mixed layer of pristine Ru-TPP and planarized Ru-TPP derivatives on Ag(111) was prepared by deposition of submonolayer coverage of Ru-TPP to a submonolayer coverage of planarized Ru-TPP derivatives on Ag(111).
CO was dosed via leak valves into the chamber, with the surface held at 150-200 K (high resolution STM / nc-AFM: 5 K).

The high resolution STM and AFM images of the planarized Ru-TPP derivatives were taken by a commercial nc-AFM/STM (CreaTec) operated at 5 K. CO was dosed on the sample surface to functionalise the tip for high resolution. A qPlus tuning fork sensor operating in the frequency modulation mode (resonance frequency ~22.6 kHz, oscillation amplitude 80 pm, Q value ~ 75000, stiffness k = 1800 N/m) without sample bias was used for nc-AFM imaging. The orientation of the Ag(111) lattice was deduced by atomically resolved STM images.

The STM images of Ru-TPP at 5 K were acquired with a different commercial low temperature STM (CreaTec).

STM images at 150-300 K were acquired by an Aarhus-type STM (SPECS GmbH).

All three STM instruments employed were housed in separate home-build UHV systems equipped with adjacent preparation chambers, employed chemically etched W tips, and were operated in constant current mode and the tunnelling bias was applied to the sample.

X-ray standing wave data were acquired at the I09 beam line at the Diamond Light Source. Measurements were performed with the sample cooled down to ~200 K. A Scienta EW4000 HAXPES analyser was used, mounted perpendicular to the incident X-rays in the horizontal plane of the photon linear polarisation. The energy of the X-rays was set to $h\nu$ = 2.63 keV (normal incidence Bragg energy). All measurements were repeated multiple times at different spots of the sample, where at each spot the reflectivity curve was measured to allow a precise energy alignment of the individual NIXSW measurements and to ensure the crystalline quality of the Ag(111). The intensity of the X-ray beam was reduced to avoid beam damage and X-ray induced CO desorption, monitored by XP spectra recorded before and after each NIXSW measurement. The desorption of CO could not entirely be avoided, which was compensated in the fitting of the Ru $3d_{5/2}$ data by a linear correction of the intensity. The correction was quantified by XP spectra of the Ru $3d_{5/2}$ region before and after each measurement, where the contributions from each species can be easily separated due to the significant difference in binding energy.

Sample preparation XPS/UPS/TPD measurements

All XPS, UPS and TPD measurements were performed in a home-built UHV chamber. The Ag(111) crystal was prepared by consecutive cycles of Ne^+-sputtering and annealing. The sample is placed on a manipulator that was cooled with LN_2. Higher temperatures required radiative counter-heating by the W filament at the back of the Ag(111) crystal. The temperature of the sample was measured *via* a K-type thermocouple junction, which is in direct contact with the Ag(111) crystal, and controlled *via* a proportional-integral-derivative (PID) controller (Schlichting Physikalische Instrumente HS 130).

Ru(CO)-TPP was sublimed from a home-built molecular evaporator at a temperature of 550-560 K, achieving deposition rates of 2 - 3 molecules/($nm^2 \cdot h$) due to the short distance between evaporator and Ag(111) crystal.

The square phase of Ru-TPP was prepared by dosing ~0.5 molecules/nm2 of Ru(CO)-TPP on the Ag(111) held at room temperature. No product of intramolecular Ru-TPP cyclodehydrogenation reactions has been observed in STM investigations after deposition of Ru-TPP on Ag(111) held at room temperature.[1-2] The Ru-TPPpl layer was prepared by annealing 10 min at 620 K. As shown by STM and nc-AFM, this annealing treatment of Ru-TPP on Ag(111) results in the complete transformation of Ru-TPP to the Ru-TPP_{pl} products **1**, **2**, **3**, and **4**.[1-2] The Ru(CO)-TPP multilayer was prepared by dosing ~1.2 molecules/nm^2 of Ru(CO)-TPP on the Ag(111) held at room temperature.

CO dosing was performed by placing the sample at 200 K in front of a needle-doser, which is attached to a differentially pumped gas dosing system. The CO exposure is monitored by a membrane gauge.

For XPS, a SPECS Phoibos 100 CCD hemispherical analyser was used to record the spectra. The sample was irradiated by non-monochromatised Mg Kα radiation (hν = 1253.6 eV) originating from a standard X-ray tube. All spectra were acquired in normal emission geometry, the energy scale was calibrated by the Ag $3d_{5/2}$ core level at 368.27 eV. XP spectra of the pristine Ru-TPP monolayer and the multilayer were acquired at 300 K, XP spectra of Ru(CO)TPP were acquired at 80 K to prevent CO desorption.

UPS was acquired using the same analyser with UV radiation from a commercial He discharge lamp (Omicron HIS 13) fitted with a linear polariser. All spectra shown in Figure 6B were taken in normal emission geometry with mixed s/p polarisation.

TPD measurements were acquired in a custom-made setup, consisting of a quadrupole mass spectrometer behind a LN_2 cooled copper cap[3] with integrated Ti sublimation pump and an aperture facing the sample. The Ag(111) crystal is placed at a close distance (~1-2 mm) in front of the cap. A camera is used to ensure the same positioning of the sample for each measurement.

DFT calculations

The DFT geometry optimization was performed using the Quantum ESPRESSO package.[4] Five layers of the silver substrate were considered within the vdW-DF2-B86r approximation[5] in the exchange-correlation term, with the two lower layers fixed at their bulk-terminated positions. An optimized lattice constant of 4.1325 Å, 2 × 2 *k* points, Fermi–Dirac smearing of occupation numbers with a 50 meV broadening, projector augmented wave datasets[6] for the pseudization of the core electrons, surface–dipole corrections, and cutoff energy of 60 Ry for the wave functions and 350 Ry for the electron density (600 Ry for the analysis) were applied. For

Ru(CO)-TPP optimization, the unit cell of Ru-TPP on Ag(111) was used, containing two molecules.[1] For the planarized Ru-TPP derivative, a unit cell of the same size with one isolated molecule was used.

Results

CO ligation on Ru-TPP at 5 K

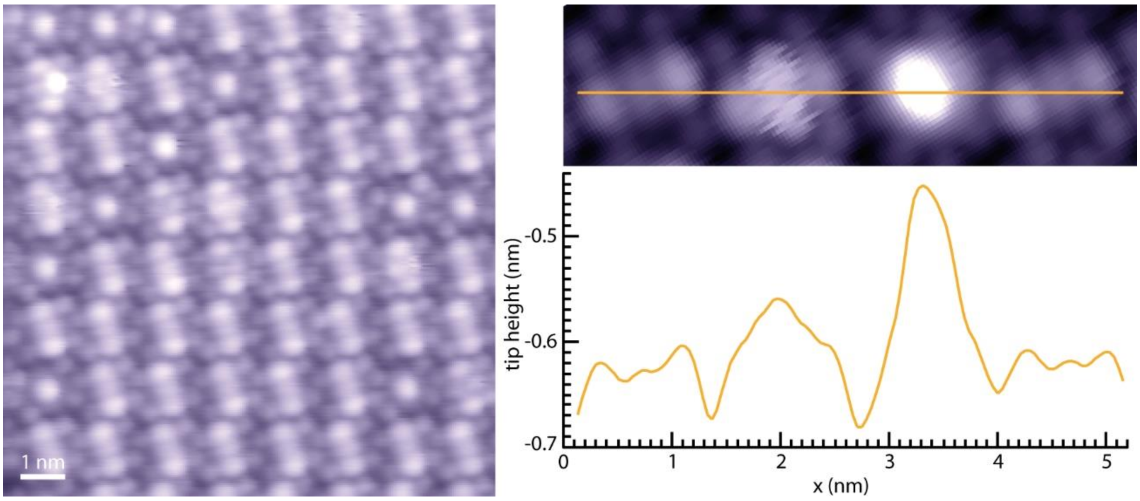

Figure S1. Left: STM image (-0.9 V, 75 pA, 5 K) of Ru-TPP partially ligated with CO. In the image we can identify CO adsorbates in both rider and axial ligation. Right: STM image (-1.0 V, 50 pA, 5 K) and line profile across (left-to-right) a Ru-TPP, a $Ru(CO)_{rider}$-TPP, a $Ru(CO)_{axial}$-TPP and a Ru-TPP molecule on Ag(111).

Cis-μ-carbonyl bonding

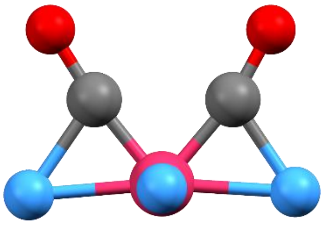

Figure S2. Model of the *cis*-μ-dicarbonyl ligation mode of CO on Ru-TPP. C, N, O, and Ru atoms are depicted in gray, blue, red, and raspberry, respectively. Adapted from [7].

STM tip manipulation for non-local CO desorption

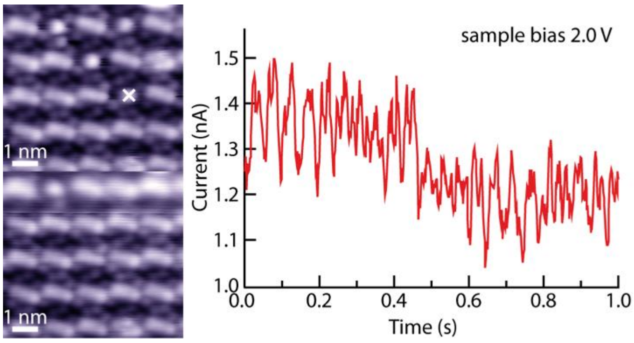

Figure S3. Non-local STM tip manipulation. The STM images (-0.9 V, 80 pA, 5 K) were acquired before (top) and after (bottom) the tip manipulation. Axially ligated CO molecules can be recognised by the bright round protrusion in the centre. At the position marked with a cross, the voltage was set to 2.0 V for 1 s with the feedback look turned off. The current trace during this operation is shown in the graph. The STM image after the manipulation shows the pristine Ru-TPP following the CO desorption from four molecules, three of which were axially ligated.

Planarized Ru-TPP on Ag(111)

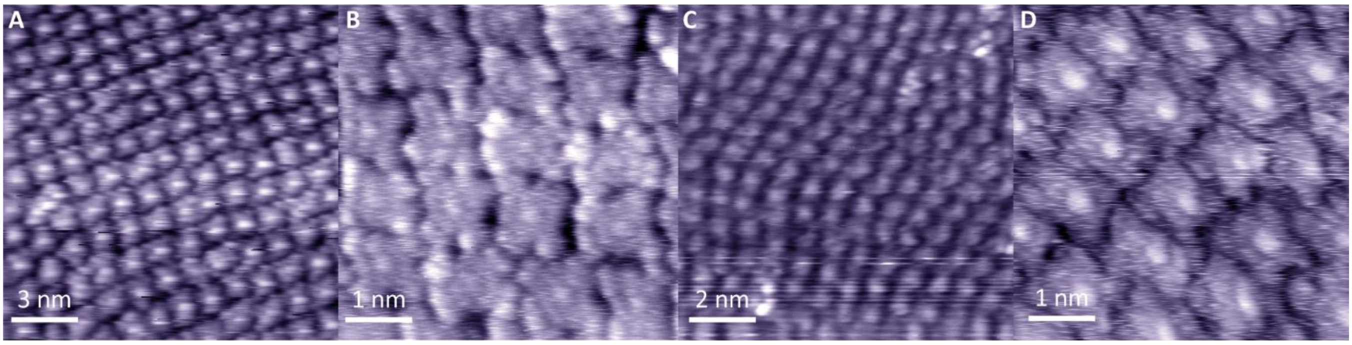

Figure S4. STM images of planarized Ru-TPP derivatives at different CO exposures (**A**: no exposure, **B**: 3 L, **C,D**: 10 L). All images were acquired at 150 K (A: -0.5 V, 100 pA, B: 0.6 V, 70 pA, C: -0.6 V, 70 pA, D: -0.5 V, 100 pA).

Mixed layer of Ru-TPP and Ru-TPP$_{pl}$ on Ag(111)

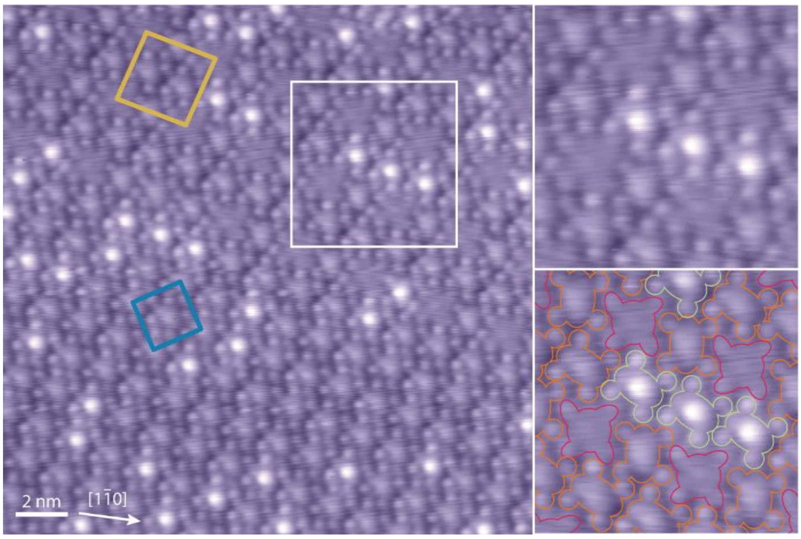

Figure S5. STM image (1.25 V, 80 pA, 150 K) of a layer of Ru-TPP and Ru-TPP$_{pl}$. Some of the Ru-TPP molecules host an axial CO ligand. The unit cell for the Ru-TPP square phase lattice is marked in blue, for the mixed Ru-TPP/Ru-TPP$_{pl}$ lattice in orange. The area shown in the white box is magnified on the right, in the bottom image the different molecules are outlined: Ru-TPP$_{pl}$ (red), Ru-TPP (orange) and Ru(CO)-TPP (green).

2D-TPD spectra of CO desorption from Ru-TPP/Ag(111)

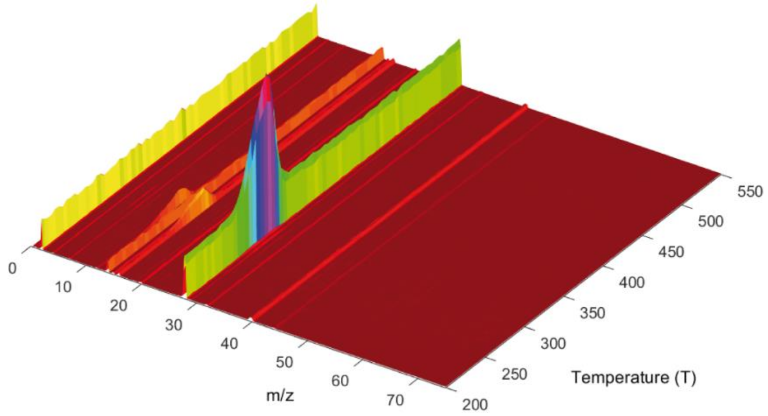

Figure S6. 2D-TPD spectra of CO desorption from the square phase of Ru-TPP/Ag(111). CO was deposited at 200 K, the heating rate was set to 2 K/s. Only peaks related to CO (m/z = 12,16,28) can be observed.

TPD analysis of CO desorption from Ru-TPP

The desorption of molecules can be described via the Polanyi-Wigner equation: $-\frac{d\theta}{dT} = \frac{v(\theta,T)}{\beta}\,\theta^n\,e^{-\frac{E_{des}(\theta,T)}{k_B T}}$ with coverage θ, order of desorption n, temperature T, frequency factor $v(\theta,T)$, heating rate β and desorption energy $E_{des}(\theta,T)$. While the heating rate is fixed (here β = 2 K/s), v and E_{des} are determined by fitting the equation to the experimental data. In principle, both parameters can be varying with temperature and coverage, but for the sake of simplicity we do not include such variations (this was found to be acceptable in the following analysis). We assume that the CO desorption follows kinetics of a first order desorption, which can be reasoned by the identical shape of the spectra for different initial coverages. Therefore, the Polanyi-Wigner equation simplifies to $-\frac{d\theta}{dT} = \frac{v}{\beta}\,\theta\,e^{-\frac{E_{des}}{k_B T}}$. Figure S8C shows the result for a single peak fit, which gives a reasonably good fit, however the frequency factor is much smaller than the expected ~10^{13} s^{-1}. By fitting two separate peaks (Figure S8A), which could be related to the different adsorption sites of the Ru (on fcc and hcp hollow sites, see Figure S7), and fixing the frequency factor to 10^{13} s^{-1}, which is commonly used for the Redhead analysis, the derived E_{des} are significantly higher, while maintaining a high fitting quality. The energetic difference between the two components amounts to 40 meV. Additional heating rate dependent data (Figure S8B) supports this fit, evident in the good agreement with the experimental data. Figure S8D shows the fitting of a single peak with a fixed $v = 10^{13}$ s^{-1}. The agreement between experimental data and fitted curve is significantly lower compared to the other two fits.

Fitting of TPD spectra for CO desorption from Ru-TPP

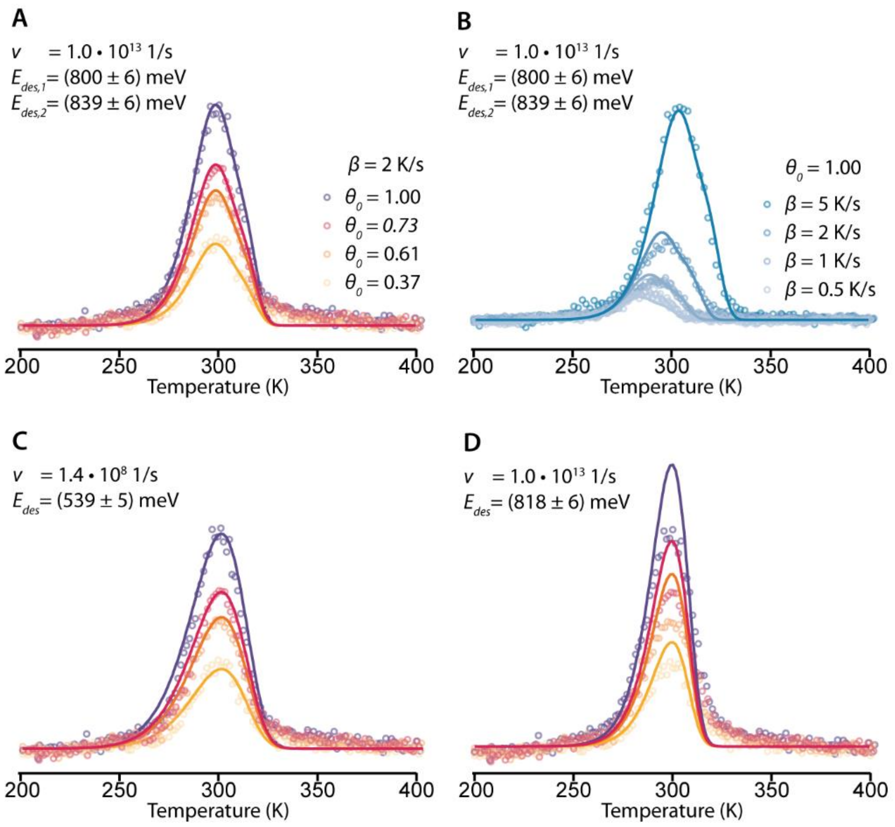

Figure S7. Fits for coverage dependant (A,C,D) and rate dependant (B) TPD spectra of CO ligation for m/z = 28 (CO). **A**,**B**: Fit with two separate peaks, *v* is fixed to 10^{13} s^{-1}. The different coverages/heating rates are given on each graph. **C**,**D**: Fit with a single peak, with v as an additional fitting parameter (C) or fixed to 10^{13} s^{-1} (D). The uncertainty of the desorption energies is estimated by the accuracy of the temperature measurement via the thermocouple, under the condition that v does not change.

DFT model of Ru(CO)-TPP on Ag(111)

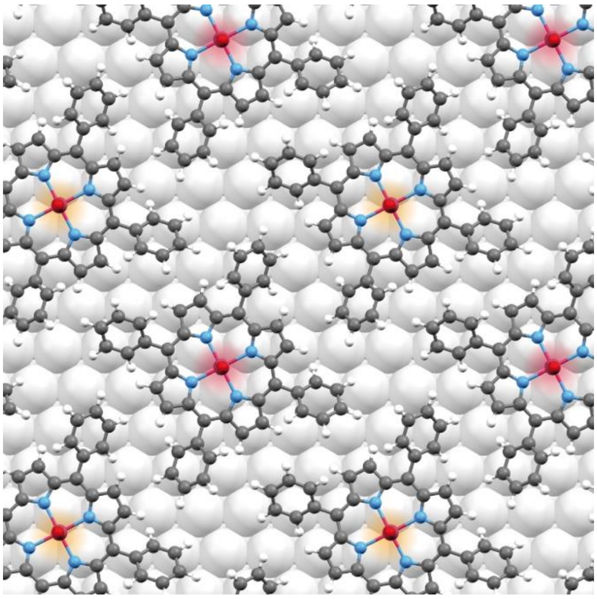

Figure S8. Top view of the Ru(CO)-TPP square phase on Ag(111) derived from the DFT energy optimisation. The two different adsorption sites of the Ru(CO) are marked in yellow (close to hcp hollow) and pink (close to fcc hollow), respectively. Ru, Ag, C, N, O, and H atoms are depicted in raspberry, silver, grey, blue, red, and white, respectively.

Density of states of the Ru centre for Ru-TPP, Ru-TPP$_{pl}$ and Ru(CO)-TPP

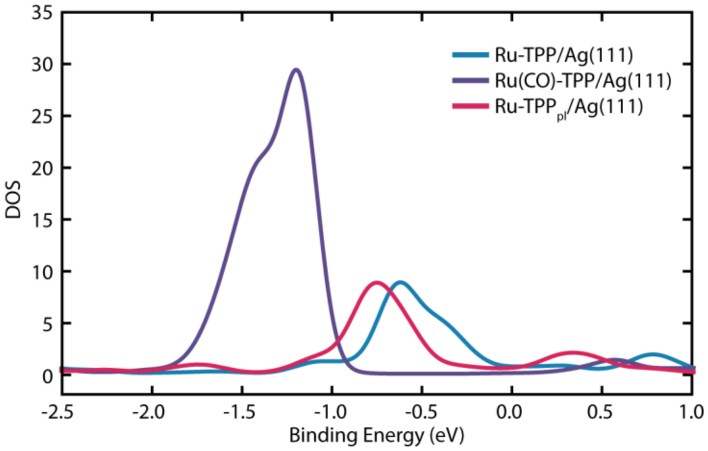

Figure S9. Projected density of states on the Ru centre, extracted from DFT, for Ru-TPP, Ru(CO)-TPP and Ru-TPP$_{pl}$ (derivative 3 in Figure 4A).

DFT model of Ru(CO)-TPP$_{pl}$

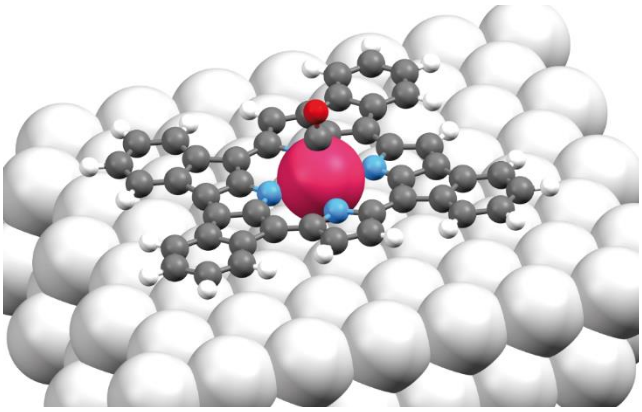

Figure S10. DFT model of Ru(CO)-TPP$_{pl}$. C, N, Ru, O, H, and Ag atoms are depicted in grey, blue, raspberry, red, white, and silver, respectively.

References

[1] P. Knecht, P. T. P. Ryan, D. A. Duncan, L. Jiang, J. Reichert, P. S. Deimel, F. Haag, J. Küchle, F. Allegretti, M. Schwarz, M. Garnica, W. Auwärter, A. P. Seitsonen, J. V. Barth, A. C. Papageorgiou, *J. Phys. Chem. C* **2021**, *125*, 3215-3224.

[2] A. Wiengarten, J. A. Lloyd, K. Seufert, J. Reichert, W. Auwärter, R. Han, D. A. Duncan, F. Allegretti, S. Fischer, S. C. Oh, Ö. Sağlam, L. Jiang, S. Vijayaraghavan, D. Écija, A. C. Papageorgiou, J. V. Barth, *Chem. - Eur. J.* **2015**, *21*, 12285-12290.

[3] a) P. Feulner, D. Menzel, *J. Vac. Sci. Technol.* **1980**, *17*, 662-663; b) S. P. Frigo, P. Feulner, B. Kassühlke, C. Keller, D. Menzel, *Phys. Rev. Lett.* **1998**, *80*, 2813-2816.

[4] P. Giannozzi, S. Baroni, N. Bonini, M. Calandra, R. Car, C. Cavazzoni, D. Ceresoli, G. L. Chiarotti, M. Cococcioni, I. Dabo, A. Dal Corso, S. de Gironcoli, S. Fabris, G. Fratesi, R. Gebauer, U. Gerstmann, C. Gougoussis, A. Kokalj, M. Lazzeri, L. Martin-Samos, N. Marzari, F. Mauri, R. Mazzarello, S. Paolini, A. Pasquarello, L. Paulatto, C. Sbraccia, S. Scandolo, G. Sclauzero, A. P. Seitsonen, A. Smogunov, P. Umari, R. M. Wentzcovitch, *J. Phys.: Condens. Matter* **2009**, *21*, 395502.

[5] a) I. Hamada, *Phys. Rev. B* **2014**, *89*, 121103; b) K. Lee, É. D. Murray, L. Kong, B. I. Lundqvist, D. C. Langreth, *Phys. Rev. B* **2010**, *82*, 081101.

[6] P. E. Blöchl, O. Jepsen, O. K. Andersen, *Phys. Rev. B* **1994**, *49*, 16223-16233.

[7] K. Seufert, M.-L. Bocquet, W. Auwärter, A. Weber-Bargioni, J. Reichert, N. Lorente, J. V. Barth, *Nat. Chem.* **2011**, *3*, 114-119.
